# A New Kernel-Based Fuzzy Level Set Method for Automated Segmentation of Medical Images in the Presence of Intensity Inhomogeneity

**DOI:** 10.1155/2014/978373

**Published:** 2014-01-29

**Authors:** Maryam Rastgarpour, Jamshid Shanbehzadeh

**Affiliations:** ^1^Department of Computer Engineering, Faculty of Engineering, Science and Research Branch, Islamic Azad University, P.O. Box 14515/775, Tehran 1477893855, Iran; ^2^Department of Computer Engineering, Faculty of Engineering, Kharazmi University, Tehran 14911-15719, Iran

## Abstract

Researchers recently apply an integrative approach to automate medical image segmentation for benefiting available methods and eliminating their disadvantages. Intensity inhomogeneity is a challenging and open problem in this area, which has received less attention by this approach. It has considerable effects on segmentation accuracy. This paper proposes a new kernel-based fuzzy level set algorithm by an integrative approach to deal with this problem. It can directly evolve from the initial level set obtained by Gaussian Kernel-Based Fuzzy *C*-Means (GKFCM). The controlling parameters of level set evolution are also estimated from the results of GKFCM. Moreover the proposed algorithm is enhanced with locally regularized evolution based on an image model that describes the composition of real-world images, in which intensity inhomogeneity is assumed as a component of an image. Such improvements make level set manipulation easier and lead to more robust segmentation in intensity inhomogeneity. The proposed algorithm has valuable benefits including automation, invariant of intensity inhomogeneity, and high accuracy. Performance evaluation of the proposed algorithm was carried on medical images from different modalities. The results confirm its effectiveness for medical image segmentation.

## 1. Introduction 

There are many structures in medical images: normal and abnormal structures. Organs, bones, muscles, and fat are in the normal structures and tumors and fractures are considered in the abnormal ones. These anatomy structures are identified by segmentation of medical images.

Image segmentation is a fundamental procedure in medical image analysis to interpret medical images. Learning how to segment anatomic structures is a significant part of medical image segmentation (MIS) [1]. The MIS is not trivial because of the complexity and variability of the ROI, poor contrast and complex nature of medical images, dependency of segmentation method on imaging modality, image features and dimensions, normal anatomic variation, postsurgical anatomic variation, vague and incomplete boundaries, artifacts, noise, and intensity inhomogeneity [1–3].

In medical imaging applications, to get better segmentation performance, practical algorithms need radiologists to adjust segmentation parameters. Most computerized systems work semiautomatically or interactively because of the complexity of parameter adjustment in the MIS. So, many works have been made to make the segmentation efficient and automatic. Machine learning provides effective means for this purpose.

**Figure 1:**
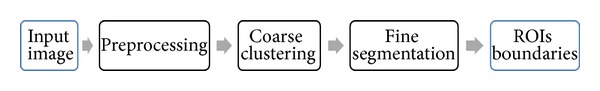
The coarse-to-fine framework of integrative approach in a hybrid intelligent system for automated image segmentation.

Some researchers [4–9] apply an integrative approach by available methods to resolve their drawbacks and enjoy their benefits along with automation.  Figure 1 shows the framework of their approach in a hybrid intelligent system for automated image segmentation. It includes two successive steps including coarse clustering and fine segmentation. For example, the authors of this paper applied the kernel-based fuzzy *c*-mean clustering algorithm [10] to overcome the dependency of initial curve in FTC model [11] in previous versions of this paper [8, 9]. Some hybrid intelligent systems have used fuzzy clustering to facilitate level set segmentation [4–9, 12]. Nevertheless, they fail in the presence of intensity inhomogeneity which often occurs in medical images.

**Figure 2:**
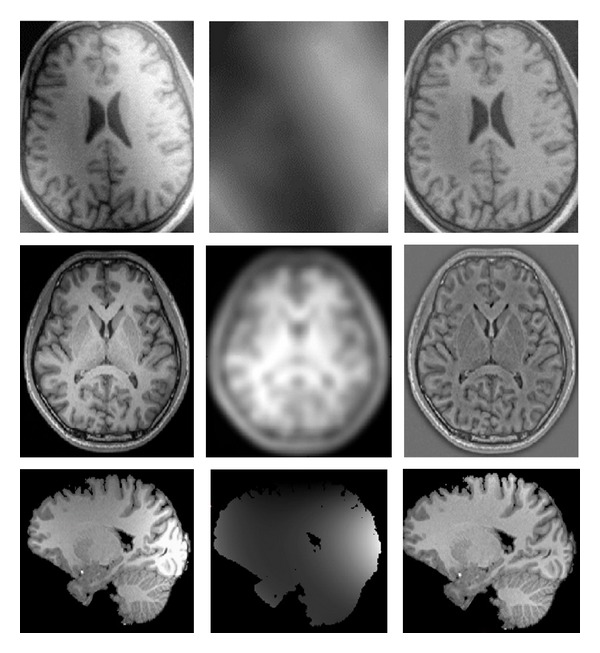
Some examples of images with intensity inhomogeneity; the columns from left to right: original images, inhomogeneity field, and corrected image; from top to bottom borrowed from [15–17], respectively.

**Figure 3:**
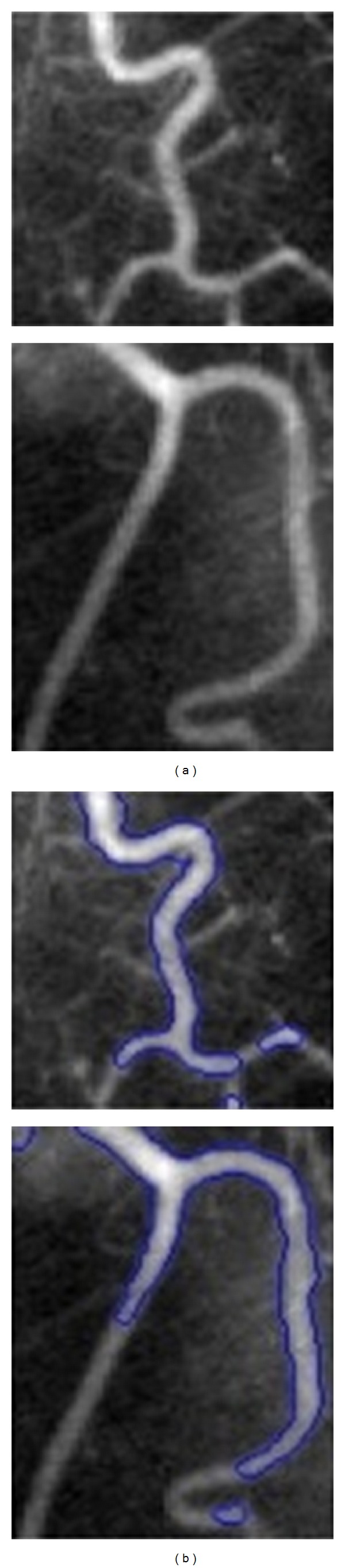
Failure of region-based level set methods for inhomogeneous images: the columns (a) original images and (b) segmentation results by blue contour [22].

Intensity inhomogeneity often arises in real images like medical images caused by spatial variations in illumination, imperfections of imaging devices, and so forth.  Figure 2 illustrates some examples of images with intensity inhomogeneity. It complicates image segmentation which is often regionbased and usually relies on the homogeneity of the image intensities in the ROI.  Figure 3 shows two samples of this failure. The reason of complexity is the overlaps between the ranges of the intensities in the regions to be segmented. This makes it impossible to identify these regions based on the pixel intensity. Vovk et al. [14] have reviewed the methods of intensity inhomogeneity correction in MRIs.

Our previous works [8, 9] have shown promising result on several types of images without the intensity inhomogeneity. It not only relieves manual intervention but also accelerates level set optimization. In this paper, we propose a new kernel-based fuzzy level set for automated medical image segmentation in the presence of intensity inhomogeneity, which has not been paid attention by our previous work and the similar ones [4–9]. Although some researchers such as [15, 16, 18–21] have investigated the segmentation problem in the images with intensity inhomogeneity recently, none of them is automatic.

The new algorithm proposed in this paper is significantly improved in the following aspects. Firstly, the GKFCM clustering can automatically estimate the parameter based on the data. Secondly, the controlling parameters of level set segmentation are now derived from the result of GKFCM directly. Thirdly, a new strategy, directed by GKFCM, is proposed to regularize level set evolution, which is different from other methods [4–9]. Fourthly, the new kernel-based fuzzy level set shows promising result in the presence of intensity inhomogeneity while the similar methods [4–9] fail. Finally, we also verified the new kernel-based fuzzy level set on general medical images with different modalities like X-ray, MRI, and CT.

The remainder of this paper is organized as follows. The next section describes the materials and methods. It first explains the kernel-based fuzzy clustering and then elaborates on level set segmentation in intensity inhomogeneity condition. It also clarifies the new kernel-based fuzzy level set algorithm in detail.  Section 3 reports our experiments and Section 4 explains the relevant discussion.  Section 5 presents concluding remarks.

## 2. Materials and Methods

### 2.1. Kernel-Based Fuzzy Clustering and Image Segmentation

The objectives of clustering algorithms overlap image segmentation problems. So, medical image segmentation problems directly apply cluster analysis developed in machine learning and pattern recognition area such as [23–26].

In fuzzy clustering, the centroid and the scope of each subclass are estimated adaptively to minimize a predefined cost function like (1) for Fuzzy *C*-Means (FCM). FCM is one of the most popular algorithms in fuzzy clustering, which has been widely applied to medical image segmentation problems. It attempts to minimize the cost function

(1) $$\begin{array}{c} J_{m}\left(U,V\right)=\sum_{i=1}^{c}\;\sum_{k=1}^{n}u_{ik}^{m}{||x_{k}-v_{i}||}^{2}, \end{array}$$

where for the MIS *x*_*k*_ is an image pixel from a dataset *X* = {*x*_1_, *x*_2_,…, *x*_*n*_} ⊂ *R* ^*P*^ (*P*, the dimension); *c* is the number of clusters and determined by a prior knowledge, that is, *c* = 4 for brain image; *n* is the number of data points; *u*_*ik*_ is the fuzzy membership of *x*_*k*_ in class *i*; *m* is a weighting exponent on each fuzzy membership and controls clustering fuzziness (usually *m* = 2); and *V* is the set of cluster centers or prototypes *v*_*i*_ ∈ *R* ^*P*^. It should be noted that *u*_*ik*_ is a member of [0, 1] and must satisfy ∑_*i*=1_^*c*^ *u*_*ik*_ = 1 and 0 < ∑_*k*=1_^*n*^ *u*_*ik*_ < *n*.

FCM can be robust to noise and outliers when replacing a new kernel-based metric in the original Euclidean norm metric of FCM. Zhang et al. [10, 27] proposed and called it kernel-based fuzzy *c*-means (KFCM) with strong noise robustness for image segmentation. The reason is that an exponential-type distance is bounded and monotone increasing, based on the concept of machine learning with a learning capability to improve the performance of clustering results [28]. The KFCM partitions a dataset *X* = {*x*_1_, *x*_2_,…, *x*_*n*_} ⊂ *R* ^*P*^, where *P* is the dimension, into *c* fuzzy subsets by minimizing the objective function:

(2) $$\begin{array}{c} J_{m}\left(U,V\right)=\sum_{i=1}^{c}\;\sum_{k=1}^{n}u_{ik}^{m}{||\Phi \left(x_{k}\right)-\Phi \left(v_{i}\right)||}^{2}, \end{array}$$

where Φ is an implicit nonlinear map and other components are the same with (1). In feature space, a kernel can be a function which is called *K*, where *K*(*x*, *y*) = 〈Φ(*x*), Φ(*y*)〉 and 〈·〉 is the inner product. Moreover, by considering the most popular kernel, that is, Gaussian radial basis function (GRBF) kernel *K*(*x*, *y*) = exp⁡⁡(−||*x*−*y*||^2^/*σ* ^2^), where *σ* is the width parameter, the objective function will be

(3) $$\begin{array}{c} J_{m}\left(U,V\right)=2\sum_{i=1}^{c}\sum_{k=1}^{n}u_{ik}^{m}\left[1-K\left(x_{k},v_{i}\right)\right]. \end{array}$$

KFCM should adjust some parameters like *σ* as dispersion. This parameter affects KFCM results. So, Yang and Tsai [28] proposed Gaussian Kernel-Based Fuzzy *C*-Means (GKFCM) clustering to estimate the parameter *σ* automatically. GKFCM can learn the other parameters by a prototype-driven learning scheme. There is no need to select the parameters in advance with prior knowledge. Moreover, it is slightly faster than KFCM. The advantage of GKFCM is to perform clustering and to estimate parameter simultaneously. The GKFCM algorithm is in Algorithm 1.

As this paper proposes a new kernel-based level set algorithm, the next subsection elaborates on level set segmentation in the presence of intensity inhomogeneity.

### 2.2. Level Set Segmentation in Intensity Inhomogeneity

Level set methods apply dynamic variational boundaries for image segmentation in contrast to the GKFCM clustering using pixel classification [13]. Level-set-based segmentation methods provide a natural and flexible way to handle many radiology images in which objects to be segmented have irregular shapes and complicated topologies [29, 30].

Level-set-based segmentation methods are generally classified into two classes: edge based and region based. Region based methods [31, 32] perform better than those based on the edge because they are less susceptible to noise and carry out more precisely in the weak edges of objects. They try to identify each ROI using a certain region descriptor such as intensity mean or a Gaussian distribution to move the active contour.

Intensity inhomogeneity affects efficiency of region-based level set segmentation methods. Moreover, defining a region descriptor for inhomogeneous images is very difficult. Li et al. [34] have proposed the local binary fitting (LBF) model to embed local image information to investigate intensity inhomogeneity in the segmentation. The basic idea is to reduce a kernel function to the LBF energy functional. In recent version of LBF model, they [35] proposed the level set evolution with bias field estimation (LSEBFE) model. They considered the model of images from the physics of imaging in a variety of modalities as *I* = *bJ* + *n*, where *J* is the true image, *b* is the component that accounts for the intensity inhomogeneity (or bias field), and *n* is additive noise. Then in view of the image model,

(4) $$\begin{array}{c} I\left(x\right)=b\left(y\right)c_{i}+n\left(x\right)\;i=1,2, \end{array}$$

where *n*(*x*) is additive zero-mean Gaussian noise and *c*_*i*_ is a constant value to approximate the intensities inside and outside the curve *C*.

By casting the segmentation problem into a higher dimensional space, the motion of the hypersurface *φ*(*t*, *x*, *y*) under the control of a speed function *F* will cause the initial boundary *φ*_0_(*x*, *y*) to move continuously till evolution. Evolving of the hypersurface can be stopped at the object boundary using image information such as edges and grey value [29]. In practice, numerical level set equation determines the evolution of *φ*(*t*, *x*, *y*):

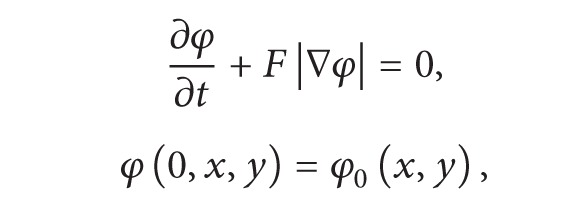
(5)

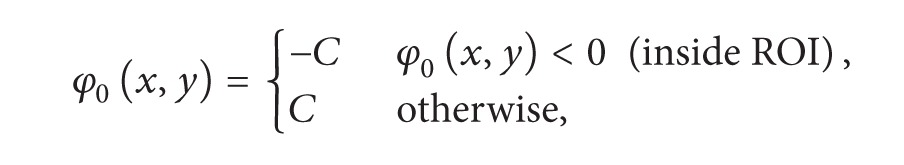
(6)
where |∇*φ*| denotes the normal direction, *φ*_0_ is the initial contour, *C* is a customable constant, and *F* represents the comprehensive forces, including the internal force (from the interface geometry such as mean curvature, contour length, and area) and external force (from image gradient and/or artificial momentums) [36]. So, the speed function *F* is

(7) $$\begin{array}{c} F\left(\varphi \right)=\nu ℰ\left(\varphi \right)+\lambda ℒ\left(\varphi \right)+\mu {ℛ}_{p}\left(\varphi \right). \end{array}$$

The constants *μ*,  *λ*,  and *ν* control the individual contributions of these terms. The energy term *ℒ*(*φ*) is the *smoothing term.* It forces *φ* to be smooth within each of the separated regions. It is also the length of zero level curve of *φ* defined by *ℒ*(*φ*) = ∫|∇*H*(*φ*)|*dx*, where *H*(*φ*) is Heaviside function *H*(*φ*) = 1/2[1 + 2/*π*arctan(*φ*/*ε*)]. The energy term *ℛ*_*p*_(*φ*) is introduced to a *distance regularization* term by Li and others [37]. It is defined by *ℛ*_*p*_(*φ*) = ∫*p*|∇*φ*|*dx* with a potential function *p* : [0, *∞*) → *ℜ* such that *p*(*s*) ≥ *p*(1) for all *s*; that is, *s* = 1 is a minimum point of *p*. To stop level set evolution near the optimal solution, that is, ROI boundary in image segmentation, a penalty momentum of *φ* deviating from the signed distance function regularizes the advancing force *F*. By substituting (4) in the data term of the LBF model [34], the data term *ℰ*(*φ*) is as follows which forces *φ* to be close to the image *I*:

(8) ℰ(φ)=∫Ω∫inside(C)Kσ(y−x)[I(x)−b(y)c1]2dx dy+∫Ω∫outside(C)Kσ(y−x)[I(x)−b(y)c2]2dx dy,

where *K*_*σ*_(*x*) = 1/((2*π*)^*n*/2^ *σ* ^*n*^)*e* ^−|*x*|^2^/2*σ*^2^^ is a Gaussian kernel with standard deviation *σ* and *c*_1_ and *c*_2_ are two constant values which approximate local intensities inside and outside the curve:

(9) c1(x)=∫(b∗Kσ(x))[H(φ(y))·I(x)]dy∫(b2∗Kσ(x))H(φ(y))dy,c2(x)=∫(b∗Kσ(x)){[1−H(φ(y))]·I(x)}dy∫(b2∗Kσ(x))[1−H(φ(y))]dy,

where ∗ is the convolution operation, *H*(*φ*) is Heaviside function, and *K*_*σ*_ is a Gaussian kernel with standard deviation *σ* defined before. The bias field (or shading image) which is the component that accounts for the intensity inhomogeneity can be computed by

(10) $$\begin{array}{c} b\left(x\right)=\frac{\left(I\left(x\right)\cdot \left\{c_{1}\cdot H\left(\varphi \left(x\right)\right)+c_{2}\cdot \left[1-H\left(\varphi \left(x\right)\right)\right]\right\}\right)*K_{\sigma }}{\left({c_{1}}^{2}\cdot H\left(\varphi \left(x\right)\right)+{c_{2}}^{2}\cdot \left[1-H\left(\varphi \left(x\right)\right)\right]\right)*K_{\sigma }}. \end{array}$$

It should be noted that *c*_1_, *c*_2_, and *b* are obtained by calculus of variation [38]. For binary segmentation, the following equation [34] computes the data term *ℰ*(*φ*):

(11) $$\begin{array}{c} e_{i}\left(x\right)=\left\{I^{2}1_{K}-2c_{i}I\left(b*K\right)+c_{i}^{2}\left(b^{2}*K\right)\;|\;i=1,2\right\}, \end{array}$$

where ∗ is the convolution operation and 1_*K*_ is the function 1_*K*_(*x*) = ∫*K*(*y* − *x*)*dy*. It equals constant 1 everywhere except near the boundary of the image domain.

### 2.3. A New Kernel-Based Fuzzy Level Set Algorithm

Level set methods and kernel-based FCM algorithms are general-purpose computational models. By constraining them to the MIS as well as integrating, we can enjoy the specific circumstances for better performance and resolve their drawbacks. To be specific, kernel-based FCM algorithms are not accurate enough for the MIS [10] and level set methods are not automatic.

To address these problems, this paper proposes a new kernel-based fuzzy level set algorithm based on a coarse-to-fine framework (Figure 1). It applies the power of curve evolution by level set to increase the efficiency of segmentation by GKFCM clustering (Algorithm 1). It also takes the advantage of suitable parameter selecting using GKFCM clustering to automate segmentation of medical images. It starts with a GKFCM clustering, whose results are applied to initiate level set segmentation, estimate controlling parameters, and regularize level set evolution in intensity inhomogeneity. The GKFCM clustering, with the ability of selecting suitable parameters by a prototype-driven learning, can achieve good segmentation results and the best score of accuracy on medical images.

**Figure 4:**
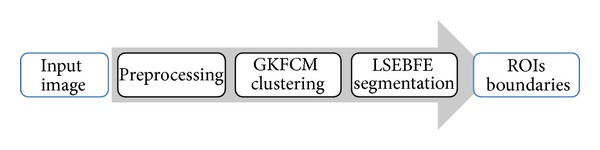
The framework of proposed approach.

Figure 4 shows the framework of proposed approach. Comparing Figures 1 and 4 shows that the proposed method applies GKFCM clustering [28] for coarse clustering and then evolves it by LSEBFE model based on an image model [35] that describes the composition of real-world images, in which intensity inhomogeneity is assumed as a component of an image.  Algorithm 2 summarizes algorithm of proposed method.

The new kernel-based fuzzy level set algorithm automates curve initialization and parameter configuration of the level set segmentation using a Gaussian kernel-based fuzzy clustering. It employs a GKFCM clustering to determine the approximate contours of interest in a medical image. Benefitting from the flexible initialization as in (6), the enhanced level set function can accommodate GKFCM results directly for evolution. So, a defuzzification process is performed to convert the fuzzy partition matrix (i.e., *U*_*MF*) to a crisp partition after converging the GKFCM algorithm. The maximum membership procedure is the most important method to defuzzify the partition matrix *U*_*MF*. This procedure assigns the pixel *i* to the class *C* with the highest membership by

(12) $$\begin{array}{c} {\;C}_{i}=\left\{\arg_{k}\left(\max\left({U\_MF}_{ki}\right)\right)\;|\;k=1,2,\ldots ,c\right\}. \end{array}$$

The proposed method applies (12) to convert the fuzzy image by the GKFCM algorithm to the crisp segmented image. It then initiates the level set as

(13) $$\begin{array}{c} \varphi_{0}\left(x,y\right)=-c_{0}\times A_{k}+\left(1-A_{k}\right)\times c_{0}, \end{array}$$

where *A*_*k*_ is a binary image obtained based on *i* = {*k* | *k* = 1,2,…, *c*} whose pixel's value is 1 if its classification is *k* and 0 otherwise, and *c*_0_ is a constant value equal to 4 in this paper. Equations (6) and (13) are the same meaning. In the experiments, we found that replacing sigma by $\sigma^{2}=\sum_{j=1}^{n}{||x_{j}-\overset{-}{x}||}^{2}/n$ with the following formula leads to better result:

(14) $$\begin{array}{c} \sigma^{2}=\frac{{\left(\max_{i=1\cdots n}\;x_{i}-\min_{i=1\cdots n}\;x_{i}\right)}^{2}}{n}. \end{array}$$

As most of the literatures note, the methods for the MIS are not general-purpose and should be configured individually. One reason is to adjust controlling parameters associated with level set methods appropriately, which varies from case to case. Li et al. [13] have listed the parameters which control level set segmentation. The new kernel-based fuzzy level set algorithm adjusts some of them based on the input image automatically. It can estimate some parameters like *σ* (by (14)) based on the input image and learn others by the prototype-driven learning scheme. Thus, it does not need to adjust all parameters in [13]. To be specific, the GKFCM clustering is robust to outliers with good parameter learning schemes. It can perform clustering and give a parameter selection simultaneously despite other clustering methods. Such methods adopt a trial-and-error technique for selecting a suitable parameter. Some researchers [13, 39–41] show some general rules for configuration of these parameters to get an optimal level set segmentation. Although it is desirable to determine these controlling parameters adaptively for the specific medical image by these useful general guidelines, they are not enough to determine the ideal configuration for a specific medical image [13].

The LSEBFE model [35] is not sensitive to the choice of the parameters. Nevertheless, the initial level set function *φ*_0_ by GKFCM clustering helps to initialize these controlling parameters and provides stable and fast evolution. On the other hand, as the zero level set from GKFCM clustering is near to the genuine boundaries, some pieces of information are adjustable like the approximate length and area of ROI. They aid to estimate some of the controlling parameters adaptively. If the ratio of area with respect to length is high, the evolution of level set will be fast. The reason is low topological complexity of the ROI in this case [13]. So,

(15) $$\begin{array}{c} \tau =\frac{\text{area}\left(\varphi_{0}\right)}{\text{length}\left(\varphi_{0}\right)}, \end{array}$$

where length(*φ*_0_) = ∫_*I*_ *δ*(*φ*_0_)*dx* *dy*,  area(*φ*_0_) = ∫_*I*_ *H*(*φ*_0_)*dx* *dy*, and $H\left(\varphi_{0}\right)=\{\begin{array}{cc} 1, & \varphi_{0}\geq 0\\ 0, & \text{otherwise} \end{array}$. The equation *μ* = 0.2/*τ* assigns the time step  *μ* inspired of the thumb rules in [13, 39–41] (i.e., *μ* × *τ* < 0.25) for stable evolution. There is no need to adjust a large *λ* to control topological changes because the zero level set by GKFCM is near to the genuine boundaries. So, we can consider *λ* = 1/*τ*.

Li et al. [13] proposed the new formula for *ν* based on the zero level set obtained SFCM to pull or push the dynamic interface adaptively toward the ROI. It has several practical benefits such as deriving from the coarse clustering SFCM directly, automatic stabilization, and the flexible selection of iteration of evolution avoiding insufficient or excessive segmentation. All mentioned benefits in [13] can be achieved in this paper implicitly due to the localization property of the Gaussian kernel function used in (13). To be specific, the contribution of the intensity *I*(*y*) to the fitting energy decreases and approaches to zero as the point *y* goes away from the center point *x*. So, the energy is dominated by the intensities *I*(*y*) of the points*y* in a neighborhood of *x*. The Gaussian kernel decreases drastically to zero as *y* goes away from *x*. In this sense, we consider that the fitting energy is localized around the point *x* [34]. Roughly speaking, the contour evolves in the narrow band of initial level set obtained by the GKFCM clustering. So, this paper considers *ν* = 1 and no need to be based on the initial level set by the GKFCM clustering in this paper.

## 3. Results

The experiments and performance evaluation were performed on medical images including a CT image of the blood vessels [34], nucleus fluorescence micrograph [42], MR image of brain [43], MR image of breast, and CT image of heart [35]. The GKFCM clustering and the proposed kernel-based fuzzy level set method were implemented with Matlab R2008a (Math Works, Natick, MA, USA) in a Windows Vista system Home Premium, Service Pack 2. All the experiments were run on a VAIO Precision 340 computer with Intel Core 2 Duo CPU P8400 at 2.26 GHz and 2 GB RAM.

### 3.1. Usefulness of GKFCM for Curve Initialization

**Figure 5:**
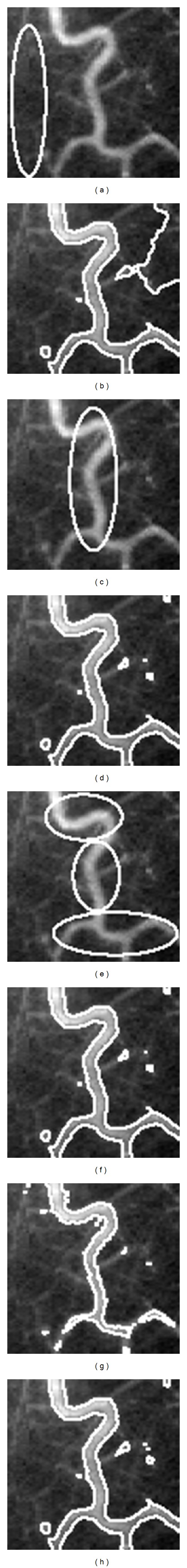
Level set segmentation of the CT vessel by various initializations: (a), (c), and (e) are manual initialization; (b), (d), (f), and (h) are final segmentation after 300, 163, 172, and 40 iterations, respectively, with *λ* = 0.003,  *μ* = 1,  and  *ν* = 1; and (g) is initialization by the GKFCM.

The first experiment assesses the GKFCM for level set initialization. It adopted the level set evolution with bias field estimation as [35] for the curve optimization, where the initialization was by three kinds of manual demarcation and GKFCM clustering.  Figure 5 represents the performance comparison on the CT image of blood vessel. Although the accuracy of the LSEBFE model is not dependent on the initial level set and it can attract the dynamic curve to ROI boundaries, the iteration of evolution is reduced because GKFCM clustering gives a curve near to the genuine boundaries (Figure 5(g)).

**Figure 6:**
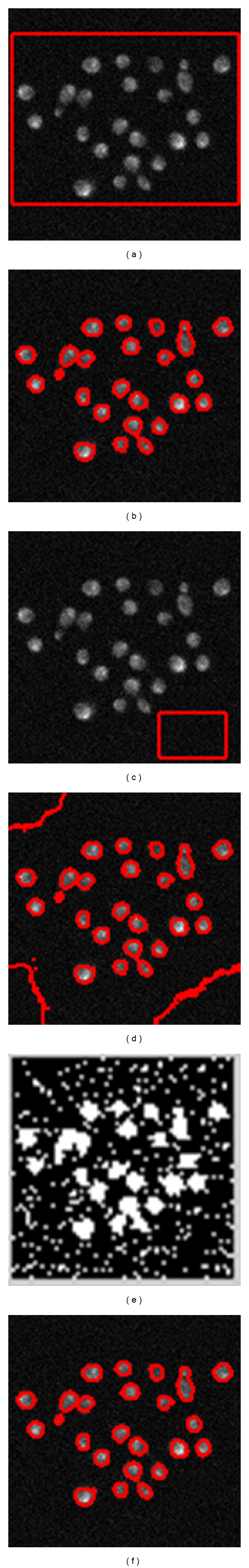
Level set segmentation of the nucleus fluorescence micrograph. (a), (c): manual initialization; (b), (d): final segmentation after 126 and 200 iterations, respectively, with *λ* = 0.003,  *μ* = 1,  and  *ν* = 1; (e): initialization by the GKFCM; and (f): final segmentation after 70 iterations.

Figure 6 illustrates result of the LSEBFE model on the nucleus fluorescence micrograph. In this case, segmentation is difficult due to the weak and irregular boundaries and inhomogeneous foreground and background. Ideal initializing is challenging again. Figure 6 proves that a GKFCM clustering has the best performance for level set initialization.

### 3.2. New Kernel-Based Fuzzy Level Set for Intensity Inhomogeneity

**Figure 7:**
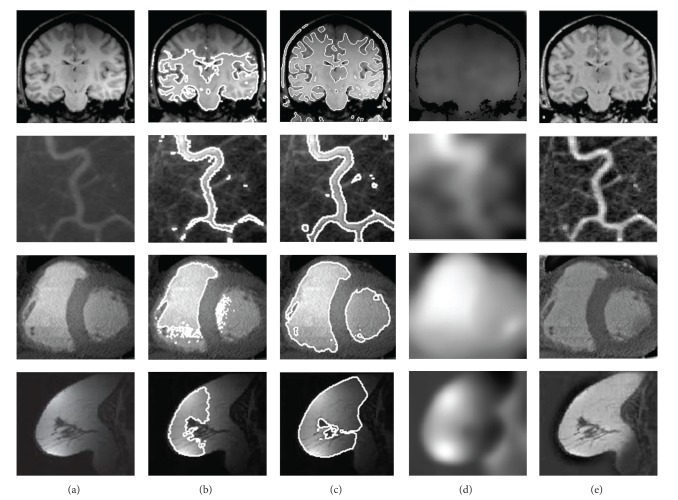
Segmentation results of various medical images by proposed method. The columns: (a) original image, (b) initial segmentation by GKFCM, (c) segmentation result, (d) bias field and (e) bias corrected image.

The second experiment evaluates the new kernel-based fuzzy level set in inhomogeneous medical images.  Figure 7 illustrates the success of new method in various modalities of medical imaging including MR images of the brain and breast (first and last rows, resp.), CT images of blood vessels and heart (second and third rows, resp.). It implicitly shows that the contour of GKFCM is near to ROI but not optimal contour of ROI.

**Figure 8:**
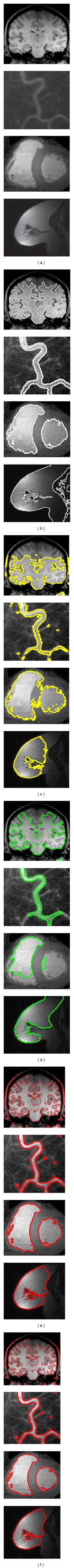
Comparison of proposed method with similar approaches [8, 13], the columns: (a) original image, (b) Ground truth in white, (c)–(f) colored segmentation result of KFCM_FTC [8], SFCM_LBF [13] and proposed method respectively.

The third experiment deals with performance evaluation and method comparison. It consists of two parts. First, the proposed method is compared with similar approaches [8, 13].  Figure 8 shows the results visually.  Table 1 presents the results quantitatively in terms of accuracy and speed, respectively.

The authors [8] recently proposed a hybrid method that initialized curve by kernel-based FCM [10] and evolved it by fast two cycle model [11]. It is referred to KFCM_FTC in this paper. B. N. Li et al. [13] have integrated spatial fuzzy *c*-means [44] with local binary fitting level set evolution [34]. It is abbreviated to SFCM_LBF henceforth.  Table 1 demonstrates that the proposed method is closer than similar approaches but it consumes more computational complexity than others.

In the second part, we used the Creaseg platform [45] and compared the segmentation result of several famous region-based level set methods [11, 31, 33] for curve evolution after initializing level set by the GKFCM clustering.

**Figure 9:**
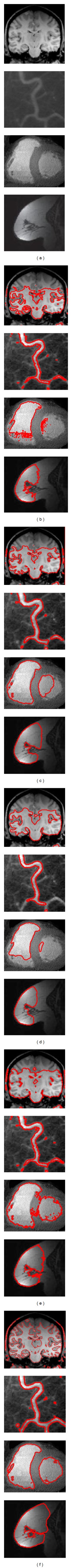
Level set segmentation of variant medical images (a) origional image, (b) initialization by GKFCM, (c) the result of CV model [31], (d) the result of localizing region-based active contours model [33], (e) the result of FTC model [11], (f) final segmentation of proposed method.

Figure 9 and Table 2 reveal this comparison qualitatively and quantitatively. Figure 9 illustrates the success of new kernel-based fuzzy level set segmentation among the famous level-set-based algorithms. In this figure, there are the original image, initialization by GKFCM, final segmentation by CV model (GKFCM_CV) [31], localizing region-based active contours (GKFCM_Lankton) [33], FTC model (GKFCM_FTC) [11], and proposed method, from left to right, respectively. Table 2 deals with this comparison in terms of speed and accuracy, respectively. Table 2 shows that the proposed method is closer than other region-based level set algorithms by spending more time (similar to part 1 of third experiment).

Dice criteria [46] calculated the similarity between the result of the algorithms and the references to get accuracy in this paper. It is popular in the segmentation problems:

(16) $$\begin{array}{c} \text{Dice}=\frac{2\left(A\cap B\right)}{A+B}, \end{array}$$

where *A* and *B* are the reference mask region and the result mask region of an algorithm.

## 4. Discussion 

The proposed method is not trivial and time consuming in medical images with somewhat clear boundaries, as seen in Figure 8, for the vessel image because it can control the motion of the level set contours in images with intensity inhomogeneity. Figures 8 and 9 illustrate this assertion by promising results of proposed method while the similar approaches failed.  Table 1 also proved this success quantitatively. Moreover, the new kernel-based fuzzy level set algorithm is able to find out the controlling parameters from the GKFCM clustering automatically.

In summary, our proposed kernel-based fuzzy level set algorithm allows flexible initialization for the MIS. One initializing paradigm was evaluated and compared in this paper in Figures 5 and 6. Manual demarcation is convenient for level set initialization and most level set systems in the literature adopt this form of initialization [45]. However, the boundaries between physiological tissues are weak and indistinct in medical images. So, manual initialization is not a reliable choice for an optimal level set segmentation with regards to image inhomogeneity and boundary leakage as shown in Figures 5 and 6.

The GKFCM clustering can adaptively get the approximate boundaries of potential components of ROI. It is also concerned with the intensity information. Thus, it is suitable to initiate level set evolution for the MIS. Level set evolution is subject to various forces from the active curve (the internal terms) and the image under investigation (the external terms). It is difficult to coordinate these forces for optimal image segmentation. Figure 9 shows that, despite good initialization, the inappropriate curve evolution may lead to an inferior segmentation.

The new kernel-based fuzzy level set algorithm is advantageous because the implicit interface stabilizes once it approaches the genuine boundaries. It is also based on an image model which is the composition of real-world images with intensity inhomogeneity as a component of an image. Besides, it is possible to estimate the nearly optimal controlling parameters from the results of the GKFCM clustering automatically. All of them facilitate the level set segmentation in practice. The kernel-based fuzzy level set method in this paper is derived from [35] where the level set evolution is subject to the intensity inhomogeneity.

It is proper to refer the work in this paper to those incorporating prior knowledge into deformable models [47]. It is not an easy task to obtain reliable prior knowledge and models in medical image analysis. The GKFCM clustering is able to obtain the potential components of ROI adaptively with the ability of parameter estimation simultaneously. It therefore serves as an effective source of prior knowledge for level set segmentation.

## 5. Conclusions 

The aim of this paper is to propose a new kernel-based fuzzy level set algorithm for automatic segmentation of medical images with intensity inhomogeneity. It employs Gaussian kernel-based fuzzy clustering as the initial level set function. It can approximate the boundaries of ROI with parameter estimation simultaneously well. So, level set evolution will start from a region close to the genuine boundaries. It also considers an image model that describes the composition of real-world images, in which intensity inhomogeneity is assumed as a component of an image. Furthermore, the new algorithm estimates the controlling parameters for curve evolution from initial level set by the GKFCM clustering automatically. This has reduced manual intervention and accelerates the curve evolution. The level set evolution stabilizes automatically once it approaches the genuine boundaries. All these improvements lead to a robust algorithm for automated medical image segmentation in the presence of intensity inhomogeneity. It also has several practical benefits such as deriving from the coarse clustering GKFCM directly, automatic stabilization, and the flexible selection of iteration of evolution avoiding insufficient or excessive segmentation. Simulation results confirm the effectiveness of proposed method for segmentation of variant medical images with intensity inhomogeneity and prove these advantages by comparing new method with several famous region-based level set segmentation methods and similar approaches.

In future research, it is interesting to incorporate simultaneously both the local spatial and the local gray level relationship in a fuzzy way for coarse clustering phase.

## Figures and Tables

**Algorithm 1:**
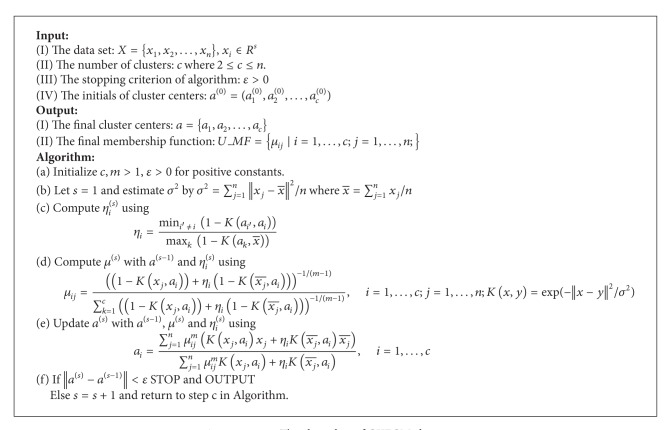
The algorithm of GKFCM clustering.

**Algorithm 2:**
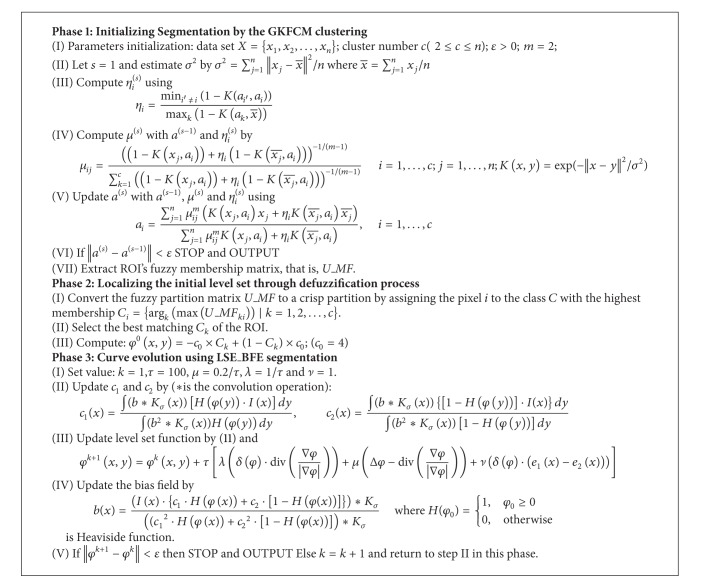
The algorithm of proposed method.

**Table 1:** Comparison of proposed method with similar approaches KFCM_FTC [8] and SFCM_LBF [13] in terms of accuracy based on Dice coefficient and CPU time in second.

Methods	Images							
	Accuracy % (Dice coefficient)				CPU time (second)			
	Brain	Vessel	Heart	Breast	Brain	Vessel	Heart	Breast
SFCM_LBF	65	75	67	82	9.2	6.55	18.94	33.93
KFCM_FTC	80	84	90	68	8.7	7.36	14.48	16.27
Proposed method	96	91	98	84	14.3	12.56	26.51	34.19

**Table 2:** Comparison of proposed method with the famous level-set-based algorithms in terms of accuracy based on Dice coefficient and CPU time in second.

Methods	Images							
	Accuracy % (Dice coefficient)				CPU time (second)			
	Brain	Vessel	Heart	Breast	Brain	Vessel	Heart	Breast
GKFCM_CV	65	85	88	64	9.6	10.49	19.42	17.36
GKFCM_Lankton	48	79	67	53	10.9	13.93	20.57	16.99
GKFCM_FTC	80	84	90	68	8.7	7.36	14.48	16.27
Proposed method	96	91	98	84	14.3	12.56	26.51	34.19
